# Association of *TYK2* polymorphisms with autoimmune diseases: A comprehensive and updated systematic review with meta-analysis

**DOI:** 10.1590/1678-4685-GMB-2020-0425

**Published:** 2021-05-03

**Authors:** Felipe Mateus Pellenz, Cristine Dieter, Natália Emerim Lemos, Andrea Carla Bauer, Bianca Marmontel de Souza, Daisy Crispim

**Affiliations:** 1 Hospital de Clínicas de Porto Alegre, Serviço de Endocrinologia, Porto Alegre, RS, Brazil.; 2 Universidade Federal do Rio Grande do Sul, Faculdade de Medicina, Programa de Pós-Graduação em Ciências Médicas, Porto Alegre, RS, Brazil.; 3 Hospital de Clínicas de Porto Alegre, Serviço de Nefrologia, Porto Alegre, RS, Brazil.

**Keywords:** Tyrosine kinase 2, autoimmunity, autoimmune disease, single nucleotide polymorphism, meta-analysis

## Abstract

Autoimmune diseases are characterized by the loss of self-tolerance, leading to immune-mediated tissue destruction and chronic inflammation. Tyrosine kinase 2 (TYK2) protein plays a key role in immunity and apoptosis pathways. Studies have reported associations between single nucleotide polymorphisms (SNPs) in the *TYK2* gene and autoimmune diseases; however, results are still inconclusive. Thus, we conducted a systematic review followed by meta-analysis. A literature search was performed to find studies that investigated associations between *TYK2* SNPs and autoimmune diseases (multiple sclerosis, systemic lupus erythematosus, Crohn’s disease, ulcerative colitis, psoriasis, rheumatoid arthritis, type 1 diabetes, and inflammatory bowel disease). Pooled odds ratios (OR) with 95 % CI were calculated using random (REM) or fixed (FEM) effects models in the Stata 11.0 Software. Thirty-four articles were eligible for inclusion in the meta-analyses, comprising 9 different SNPs: rs280496, rs280500, rs280523, rs280519, rs2304256, rs12720270, rs12720356, rs34536443, and rs35018800. Meta-analysis results showed the minor alleles of rs2304256, rs12720270, rs12720356, rs34536443, and rs35018800 SNPs were associated with protection against autoimmune diseases. Moreover, the A allele of the rs280519 SNP was associated with risk for systemic lupus erythematosus. Our meta-analyses demonstrated that the rs2304256, rs12720270, rs12720356, rs34536443, rs35018800, and rs280519 SNPs in the *TYK2* gene are associated with different autoimmune diseases.

## Introduction

Autoimmune diseases are complex diseases triggered by multifaceted interactions between several genetic and environmental factors (Gutierrez-Roelens and Lauwerys, 2008; Rose, 2016), and are characterized by the loss of self-tolerance leading to immune-mediated tissue destruction and chronic inflammation (Marrack et al., 2001; Lee and Bae, 2016; Odhams et al., 2017). These diseases share common etiological pathways, with genetic factors being considered as strong determinants of their development (Gutierrez-Roelens and Lauwerys, 2008; Lee and Bae, 2016). Regarding genetic factors, *tyrosine kinase 2* (*TYK2*) is a candidate gene for autoimmune diseases since it encodes a member of Janus Kinase (JAK) family of tyrosine kinases, which have a central role in immune response since they mediate signaling pathways for several cytokines and type I interferon (IFN-I) (Ghoreschi et al., 2009; Strobl et al., 2011).

TYK2 is a non-receptor protein that bounds to the IFN-I receptor (IFNAR1) on the cell surface in its inactive form. After IFN-α binding to IFNAR1, TYK2 and JAK1 proteins are activated, leading to the recruitment and phosphorylation of the signal of transducers and activators of transcription (STAT) 1 and 2. STAT1/2 heterodimers then translocate to the nucleus, where they are major regulators of the expression of a number of IFN-stimulated genes (Yamaoka *et al.*, 2004; Strobl et al., 2011). TYK2 is also associated with IL-6, IL-10, IL-12, and IL-23 receptors, playing a key role in the activation of these cytokine pathways (Ghoreschi et al., 2009; O’Shea and Plenge, 2012). Abnormal expression of IFN-I and other cytokines or JAK kinase members in immune cells are well known players in the pathogenesis of autoimmune diseases (Strobl *et al.*, 2011; O’Shea and Plenge, 2012; Deng et al., 2019). Besides its role in the IFN-I and other type I and II cytokine receptor pathways, TYK2 plays a key role in other immune processes, including the activity of natural killer cells, maturation of B and Treg cells, and differentiation of Th1 and Th17 cells. Accordingly, dysregulated *TYK2* expression has been associated with autoimmune diseases, specially systemic lupus erythematosus (SLE) [reviewed in (Deng *et al.*, 2019)].

Consistent with the role of TYK2 in immune processes, several studies have suggested common single nucleotide polymorphisms (SNPs) in this gene are associated with different autoimmune diseases, including multiple sclerosis (MS) (Tao et al., 2011), SLE (Tao *et al.*, 2011; Lee et al., 2012; Lee and Bae, 2016; Yin et al., 2018), Crohn’s Disease (CD) (Lees et al., 2011; Tao *et al.*, 2011; Ellinghaus et al., 2016), ulcerative colitis (UC) (Lees *et al.*, 2011; Tao *et al.*, 2011; Ellinghaus *et al.*, 2016), rheumatoid arthritis (RA) (Tao *et al.*, 2011; Lee and Bae, 2016; Westra et al., 2018), type 1 diabetes mellitus (T1DM) (Nagafuchi et al., 2015; Westra *et al.*, 2018), and psoriasis (Pso) (Ellinghaus *et al.*, 2016). In 2011, Tao *et al*. (2011) published a meta-analysis of 11 studies that investigated the association between 6 *TYK2* SNPs and autoimmune and inflammatory diseases. The authors showed an association between the *TYK2* rs2304256 and rs34536443 SNPs and MS, RA, SLE, CD, and UC. Lee and Bae (2016) performed a meta-analysis of 12 studies regarding the association of 7 *TYK2* SNPs with SLE and RA, showing the rs2304256 and rs1270356 minor alleles were associated with protection against these rheumatic diseases. Five other SNPs (rs12720270, rs280500, rs280523, rs8108236, and rs280519) were not associated with these diseases; however, the number of studies included in their meta-analyses was small. A recent meta-analysis suggested the association of the *TYK2* rs2304256 C allele with risk for SLE in Europeans (3 studies) but not in Asians (3 studies), while the rs12720270 and rs280519 SNPs were not associated with SLE (3 studies each) (Yin *et al.*, 2018). Therefore, different SNPs in the *TYK2* gene seem to be associated with autoimmune diseases, although the results on individual SNPs are still inconclusive (Tao *et al.*, 2011; Lee *et al.*, 2012; Ellinghaus *et al.*, 2016; Lee and Bae, 2016; Westra *et al.*, 2018; Yin *et al.*, 2018) especially due to the increase in the number of studies in this field in the last few years in different ethnicities. Thus, here, we performed a comprehensive and updated meta-analysis of the related literature aiming to clarify the role of different *TYK2* SNPs on susceptibility to autoimmune diseases.

## Material and Methods

### Search strategy and eligibility criteria

This systematic review was performed and described following PRISMA and MOOSE guidelines (Stroup et al., 2000; Moher et al., 2009), and its protocol was registered in the International Prospective Register of Systematic Reviews (PROSPERO) under the CRD42018100302 number. In order to identify studies that investigated associations between *TYK2* SNPs and autoimmune diseases, we performed a literature search in Embase and PubMed resources. For this, the following MeSH terms were applied: (“*TYK2* Kinase” OR “TYK2 protein, human”) AND (“Autoimmune Diseases” OR “Rheumatic Diseases” OR “Lupus Erythematosus, Systemic” OR “Multiple Sclerosis” OR “Sclerosis” OR “Crohn Disease” OR “Pediatric Crohn’s disease” OR “Ulcerative colitis” OR “Psoriasis” OR “Diabetes Mellitus” OR “Diabetes Mellitus, Type 1”). The search was completed on June, 2020, and was restricted to papers written in English, Spanish or Portuguese. Studies were also searched in the GWAS Catalog (https://www.ebi.ac.uk/gwas/).

Eligibility assessment was done by reviewing titles and abstracts of all articles selected, and when abstracts did not provide necessary information, the full text of the article was analyzed. This was performed independently, in a standardized manner, by two investigators (C.D. and F.M.P.), as previously described in other meta-analyses from our group (Souza et al., 2013; Brondani et al., 2014). Discordances were settled by debate between them and, when needed, a third investigator (D.C.) was referred. When articles had missing information, we contacted the authors for further information. In case of duplicated data that had been published more than once, we opted to include the most complete study. In addition, reference lists from all articles fulfilling the eligibility criteria were manually searched to identify other important citations.

Studies were considered eligible if they had case-control designs and evaluated one or more *TYK2* SNPs in patients with some of the autoimmune diseases included in the MESH terms (cases) and individuals without any autoimmune condition (controls). Exclusion criteria were as follows: 1) Studies that did not have sufficient data to estimate an OR with 95 % CI; and 2) Studies where genotype distributions in control group deviated from those predicted by the Hardy-Weinberg equilibrium (HWE).

### Data extraction and quality control assessment

Data were individually extracted by two researchers (C.D. and F.M.P.) using a standardized form (Souza et al., 2013; Brondani et al., 2014), and agreement was pursued in all extracted items. When an agreement could not be achieved, data extraction divergences were solved by referencing to the original publication or by consulting a third reviewer (D.C.). Data extracted from each study included: publication year, name of the first author, number of cases and controls, autoimmune disease, gender, age, ethnicity, genotyping technique, genotype and allele distributions in case and control samples and OR (95 % CI). We included in the meta-analysis only those SNPs investigated in at least 3 studies.

The Newcastle-Ottawa Scale (NOS) for case-control studies was used to analyze the quality of each eligible study (Wells et al., 2020). Two investigators (C.D. and F.M.P.) evaluated the 9 items of the NOS, which are categorized into 3 dimensions: selection, comparability, and exposure. Each item contains a sequence of alternative questions to be answered by the investigators. Then, a star scoring system allows the semi-quantitative analysis of article quality. In this score, the highest-quality studies receive one star for each item, excepting the comparability item that can receive two stars. Thus, the range of stars in the NOS score varies from zero to nine. The Clark-Baudouin Score (CBS) was also used to assess the quality of the studies (Clark and Baudouin, 2006). This method uses pre-defined criteria to assess each publication, highlighting quality issues in the conduction of studies and interpretation of the results. Using a 10-point scoring sheet, researchers are able to evaluate components of the articles related to reproducibility, selection of subjects, statistical analysis and genotyping methods.

### Statistical analysis

Goodness-of-fit χ^2^ tests were used to evaluate whether the genotype frequencies in the control groups were in agreement with those predicted by the Hardy-Weinberg Equilibrium (HWE). Gene-disease associations were measured using OR (95 % CI) calculations based on allele contrast, additive, recessive, and dominant inheritance models, which were categorized as suggested by Zintzaras et al. (2008). Stratifications by autoimmune disease type and/or ethnicity were performed when a disease / ethnicity had ≥ 2 studies for each assessed SNP. Heterogeneity among studies was evaluated using a χ^2^-based Cochran’s Q statistic and inconsistency was calculated using the I^2^ metric. When P < 0.10 for the Q test and/or I^2^ > 50 %, heterogeneity among studies was considered significant. Then, the DerSimonian and Laird random effects model (REM) was used to calculate OR (95 % CI) for each study and for the pooled effect. In the lack of significant heterogeneity, the fixed effect model (FEM) was used for this calculation (Higgins et al., 2003; Melsen et al., 2014).

In the case of relevant inter-study heterogeneity, sensitivity analyses were performed to identify which studies could have a considerable impact on heterogeneity. Risk of publication bias was evaluated for SNPs analyzed in ≥ 10 studies using funnel plot graphics, analyzed both visually and using the Begg and Egger statistic (Egger et al., 1997). The significance of the intercept was determined by the *t* test, as proposed by Egger, with P < 0.10 being considered indicative of significant publication bias (Egger *et al.*, 1997). In case of significant publication bias, the Trim and Fill method was used for adjusting for it. This method evaluates whether publication bias is present and, then, estimates the pooled effect when biases are removed (Duval and Tweedie, 2000). The Stata 11.0 software (StataCorp, College Station, TX, USA) was used for all statistical analyses.

## Results

### Results from the literature search and quality of the studies

**Figure 1:**
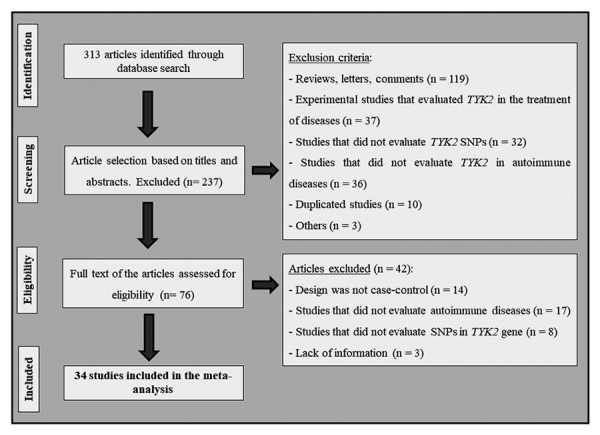
Flowchart illustrating the search strategy used to identify studies of *TYK2* SNPs and autoimmune diseases.

Figure 1 shows the flow diagram with the strategy used to identify and select studies for inclusion in this systematic review and meta-analysis. A total of 313 articles were retrieved after searching PubMed, Embase, and GWAS Catalog resources, and 237 of them were excluded following the review of titles and abstracts due to disagreements with our defined eligibility criteria. Seventy-six articles were therefore considered as being eligible at this point and had their full texts examined. Nevertheless, after analyzing the full texts, another 42 studies were excluded, and a total of 34 articles (Sigurdsson et al., 2005, 2007; International Consortium for Systemic Lupus Erythematosus Genetics *et al.*, 2008; Ban et al., 2009; Hellquist et al., 2009; Kyogoku et al., 2009; Sato et al., 2009; Suarez-Gestal et al., 2009a, b; Genetic Analysis of Psoriasis et al., 2010; Jarvinen et al., 2010; Mero et al., 2010; Couturier et al., 2011; Graham et al., 2011; Li et al., 2011; Lian et al., 2013; Qiu et al., 2013; Shaiq et al., 2013; Alonso-Perez et al., 2014; Can et al., 2015; Diogo et al., 2015; Nagafuchi et al., 2015; Prieto-Perez et al., 2015; Tang et al., 2015; Ellinghaus et al., 2016; Lopez-Isac et al., 2016; Almlof et al., 2017; Langefeld et al., 2017; Myrthianou et al., 2017; Westra et al., 2018; Zaplakhova et al., 2018; Contreras-Cubas et al., 2019; Graciolo et al., 2019; Mohamadhosseini et al., 2019) met the eligibility criteria and were included in this meta-analysis. 

Nine SNPs in the *TYK2* gene were investigated in ≥ 3 studies and then were included in our meta-analyses. Table S1 shows genotype and allele frequencies of these SNPs in case and control groups from the eligible studies as well as in which autoimmune disease and ethnicity they were analyzed. Among the 34 eligible articles, 4 studies analyzed the rs280496 (g.10352804C>G) SNP (408 cases with CD or UC / 578 controls), 6 studies, the rs280500 (g.10379726A>G) SNP (2 988 cases with SLE / 6 440 controls), 14 studies, the rs280519 (g.10362257A>C) SNP (13 969 cases with CD, UC, Pso or SLE / 29 167 controls), and 6 studies investigated the rs280523 (g.10366530G>A) SNP (997 cases with CD, UC or SLE / 955 controls). Moreover, 25 studies evaluated the rs2304256 (g.10364976C>A) SNP (23 827 cases with CD, UC, SLE, MS, RA or T1DM / 35 760 controls), 9 studies, the rs12720270 (g.10365084G>A) SNP (2 792 SLE cases / 5 184 controls), and 20 studies analyzed the rs12720356 (g.10359299A>C) SNP (69 788 cases with SLE, RA, IBD, MS, CD, UC or Pso / 177 438 controls). Nineteen studies evaluated the rs34536443 (g.10352442G>C) SNP (50 011 cases with MS, RA, SLE, IBD, Pso or T1DM / 95 923 controls), while 8 studies analyzed the rs35018800 (g.10354167G>A) SNP (61 241 cases with RA, SLE, IBD, CD, Pso, UC or MS / 163 386 controls).

The quality of each individual study is shown in Table S2. As mentioned in the Material and Methods section, the highest quality articles can receive up to 9 stars for the NOS score. Most of the included studies were classified as presenting good quality since 61.8 % of the studies were awarded 6 to 8 stars and 38.2 % of the studies received the maximum of stars allowed. None of the articles scored less than 6 stars. Regarding the CBS score, most of the studies were also classified as presenting good quality since 73.5 % of them received 7 to 9 points and 26.5 % received 10 points, which is the highest score.

### Meta-analyses of studies that evaluated associations between TYK2 SNPs and autoimmune diseases

Table 1 shows results of the pooled analyses for the associations between *TYK2* rs280496, rs280500, rs280519, rs280523, rs2304256, rs12720270, rs12720356, rs34536443, and rs35018800 SNPs and autoimmune diseases under different inheritance models. When the number of studies was statistically adequate, we also evaluated these associations after stratification by disease type and/or ethnicity (Table 1).

**Table 1 -:** Pooled measures for associations between *TYK2* rs280496, rs280500, rs280519, rs280523, rs2304256, rs12720270, rs12720356, rs34536443, and rs35018800 SNPs and susceptibility to autoimmune diseases.

Inheritance model	n studies	n cases	n controls	I² %	Pooled OR (95 % CI)
***rs280496***					
Allele contrast	4	408	578	0.0	1.15 (0.89 - 1.49)*
*Disease*					
CD	2	143	289	0.0	1.19 (0.80 - 1.76)*
UC	2	265	289	0.0	1.12 (0.80 - 1.58)*
Dominant	3	294	378	0.0	1.21 (0.86 - 1.70)*
***rs280500***					
Allele contrast	6	2 988	6 440	55.7	1.12 (0.95 - 1.31)**
***rs280523***					
Allele contrast	6	997	955	0.0	1.11 (0.87 - 1.41)*
*Disease*					
CD	2	143	289	0.0	0.90 (0.51 - 1.57)*
UC	2	265	289	0.0	1.07 (0.68 - 1.70)*
SLE	2	589	377	0.0	1.21 (0.87 - 1.68)*
*Ethnicity*					
Asian	4	408	578	0.0	1.00 (0.70 - 1.42)*
Caucasian	2	589	377	0.0	1.21 (0.87 - 1.68)*
Dominant	3	294	378	0.0	0.95 (0.60 - 1.49)*
***rs280519***					
Allele contrast	14	13 969	29 167	87.1	1.07 (0.96 - 1.20)**
*Disease*					
CD	3	223	389	58.7	1.08 (0.74 - 1.57)**
UC	2	265	289	0.0	1.21 (0.94 - 1.55)**
SLE	8	6 733	16 973	38.5	1.10 (1.04 - 1.18)**
Pso	1	6 748	11 516	-	-
*Ethnicity*					
Asian	8	1 868	2 538	34.4	1.08 (0.95 - 1.22)**
Caucasian	6	12 101	25 245	94.2	1.07 (0.91 - 1.25)**
Recessive	6	1 085	1 184	65.6	1.18 (0.80 - 1.75)**
Dominant	6	1 085	1 184	0.0	0.84 (0.70 - 1.02)*
Additive	6	618	629	41.8	0.89 (0.71 - 1.12)*
***rs2304256***					
Allele contrast	25	23 827	35 760	70.8	0.83 (0.77 - 0.88)**
*Disease*					
SLE	12	7 315	11 736	72.9	0.77 (0.69 - 0.85)**
CD	3	223	389	53.5	0.77 (0.52 - 1.15)**
UC	2	265	289	0.0	0.84 (0.64 - 1.09)**
T1DM	3	551	573	56.6	1.03 (0.77 - 1.38)**
MS	3	12 312	20 010	0.0	0.84 (0.81 - 0.87)**
RA	2	3 161	2 763	33.3	0.99 (0.89 - 1.09)**
*Ethnicity*					
Caucasian	12	20 474	30 034	73.8	0.81 (0.75 - 0.87)**
Asian	10	2 678	4 891	74.7	0.85 (0.71 - 1.01)**
Mixed Ethnicity	3	675	835	31.6	0.87 (0.69 - 1.09)**
Recessive	13	16 916	26 506	78.3	0.80 (0.65 - 0.98)**
*Disease*					
SLE	4	3 759	5 545	88.5	0.62 (0.40 - 0.97)**
CD	2	143	289	33.8	0.88 (0.45 - 1.75)**
UC	1	151	89	-	-
T1DM	3	551	573	0.0	1.51 (0.97 - 2.34)**
MS	3	12 312	20 010	0.0	0.79 (0.73 - 0.87)**
*Ethnicity*					
Caucasian	4	14 794	24 134	83.7	0.64 (0.51 - 0.82)**
Asian	7	1 815	2 053	80.7	0.87 (0.55 - 1.37)**
Mixed Ethnicity	2	307	319	0.0	1.12 (0.54 - 2.30)**
Dominant	14	16 996	26 606	45.0	0.78 (0.72 - 0.84)**
*Disease*					
SLE	4	3 759	5 545	13.0	0.75 (0.67 - 0.83)**
CD	3	223	389	63.9	0.60 (0.31 - 1.14)**
UC	1	151	89	-	-
T1DM	3	551	573	49.8	0.93 (0.66 - 1.31)**
MS	3	12 312	20 010	0.0	0.81 (0.77 - 0.85)**
*Ethnicity*					
Caucasian	4	14 794	24 134	28.9	0.79 (0.75 - 0.83)**
Asian	8	1 895	2 153	59.0	0.72 (0.55 - 0.95)**
Mixed Ethnicity	2	307	319	56.0	0.82 (0.51 - 1.33)**
Additive	13	10 778	15 955	72.2	0.68 (0.55 - 0.84)**
*Disease*					
SLE	4	2 330	3 325	86.2	0.58 (0.34 - 0.99)**
CD	2	103	164	0.0	0.43 (0.21 - 0.88)**
UC	1	113	54	-	-
T1DM	3	345	334	0.0	1.45 (0.92 - 2.28)**
MS	3	7 887	12 078	0.0	0.73 (0.67 - 0.80)**
*Ethnicity*					
Caucasian	4	9 437	14 493	84.5	0.59 (0.46 - 0.75)**
Asian	7	1 136	1 266	68.3	0.73 (0.46 - 1.16)**
Mixed Ethnicity	2	205	196	0.0	1.03 (0.49 - 2.14)**
***rs12720356***					
Allele contrast	20	69 788	177 438	92.1	0.85 (0.77 - 0.94)**
*Disease*					
SLE	9	6 360	20 668	49.9	0.75 (0.65 - 0.88)**
RA	2	6 256	14 544	11.5	0.91 (0.83 - 1.00)**
IBD	1	1 346	13 683	-	-
CD	1	19 085	34 213	-	-
Pso	4	10 240	40 419	63.2	0.71 (0.61 - 0.83)**
UC	1	14 413	34 213	-	-
MS	2	12 088	19 698	0.0	0.85 (0.79 - 0.90)**
Recessive	4	13 437	22 606	1.1	0.85 (0.64 - 1.13)*
Dominant	4	13 437	22 606	34.2	0.82 (0.77 - 0.87)*
Additive	4	11 787	19 301	4.4	0.83 (0.62 - 1.10)*
***rs34536443***					
Allele contrast	19	50 011	95 923	75.7	0.68 (0.61 - 0.76)**
*Disease*					
MS	9	21 346	27 989	38.6	0.75 (0.67 - 0.83)**
RA	3	5 818	14 894	62.4	0.83 (0.54 - 1.29)**
SLE	4	12 041	27 735	25.4	0.50 (0.43 - 0.57)**
IBD	1	1 346	13 687	-	-
Pso	1	126	507	-	-
T1DM	1	9 334	11 111	-	-
Recessive	4	13 180	20 905	67.1	0.35 (0.03 - 3.83)**
Dominant	5	13 306	21 412	88.0	0.34 (0.21 - 0.56)**
Additive	4	12 834	20 478	67.2	0.35 (0.03 - 3.88)**
***rs12720270***					
Allele contrast	9	2 792	5 184	30.2	0.92 (0.84 - 1.00)*
*Ethnicity*					
Asian	3	1 380	3 274	0.0	0.92 (0.87 - 1.08)*
Caucasian	5	1 044	1 394	51.7	0.84 (0.72 - 0.98)*
Mixed Ethnicity	1	368	516	-	-
***rs35018800***					
Allele contrast	8	61 241	163 386	18.0	0.60 (0.55 - 0.65)*

Where significant heterogeneity was detected (I^2^ > 50 % and/or Q statistic P>0.1), the DerSimonian and Laird random effect model (REM)** was used to calculate OR (95 % CI) for each individual study and for the pooled effect; where heterogeneity was not significant, the fixed effect model (FEM)* was used for this calculation. CD: Crohn’s disease; IBD: inflammatory bowel disease; MS: multiple sclerosis; Pso: psoriasis; RA: rheumatoid arthritis; SLE: systemic lupus erythematosus; T1DM: type 1 diabetes mellitus; UC: ulcerative colitis.

Our results show the *TYK2* rs280496, rs280500, and rs280523 SNPs were not associated with autoimmune diseases under an allele contrast model (Table 1 and Figures S1 and S2). Overall, the rs280519 SNP was also not associated with autoimmune diseases considering allele, dominant, recessive, and additive models (Table 1 and Figure S2). However, after stratification by disease type, the rs280519 SNP was independently associated with risk for SLE (REM OR 1.10, 95 % CI 1.04 - 1.18, allele contrast model; Table 1 and Figure S2). 

**Figure 2 -:**
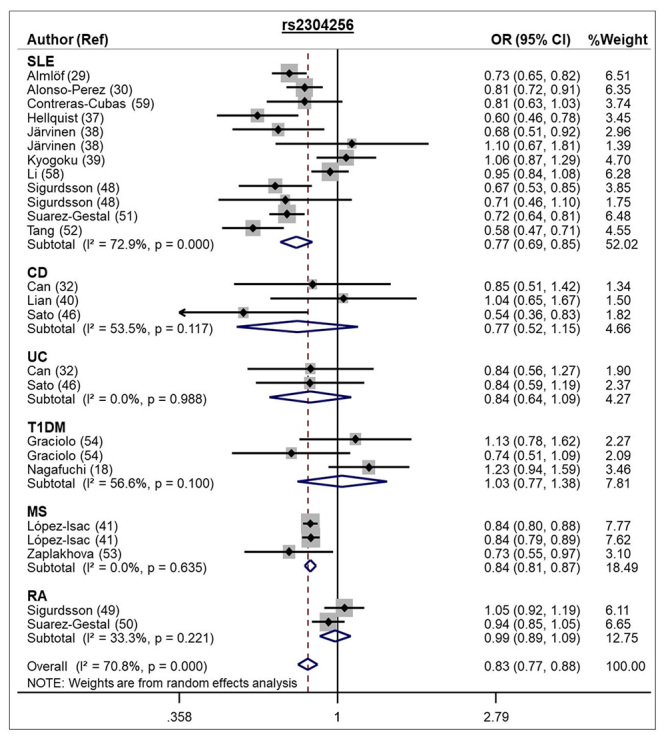
Forest plot showing individual and pooled OR (95 % CI) for the association between the *TYK2* rs2304256 SNP and autoimmune diseases, under an allele contrast model.

**Figure 3 -:**
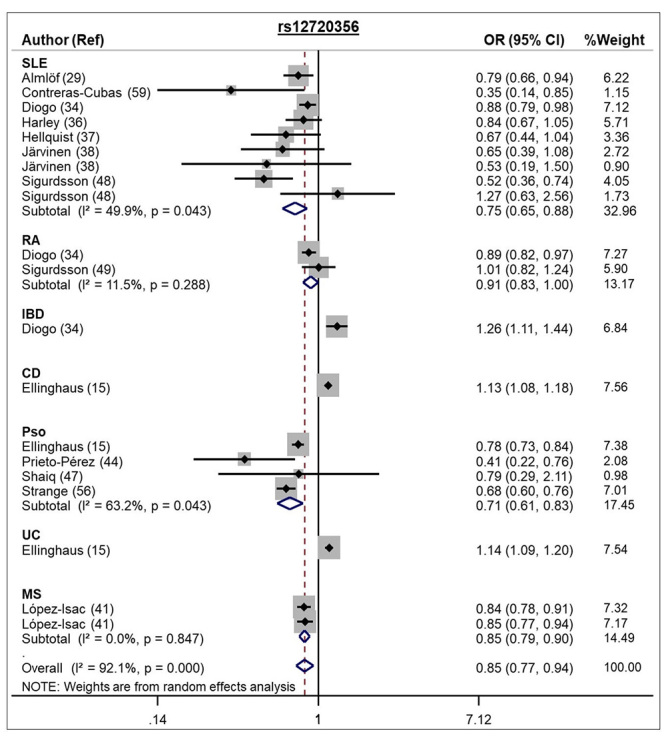
Forest plot showing individual and pooled OR (95 % CI) for the associations between the *TYK2* rs12720356 SNP and autoimmune diseases, under an allele contrast model.

**Figure 4 -:**
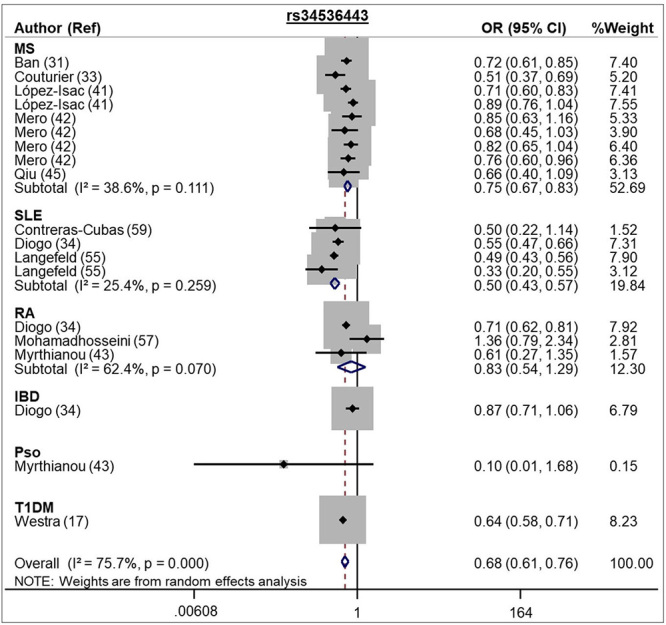
Forest plot showing individual and pooled OR (95 % CI) for the associations between the *TYK2* rs34536443 SNP and autoimmune diseases, under an allele contrast model.

The A allele of the rs2304256 SNP was associated with protection against autoimmune diseases when assuming the allele contrast model (REM OR 0.83, 95 % CI 0.77 - 0.88; Table 1 and Figure 2). This SNP was also significantly associated with autoimmune diseases under dominant, recessive, and additive models (Table 1). In addition, the C allele of the rs12720356 SNP conferred protection against different autoimmune diseases under both the allele contrast (REM OR 0.85, 95 % CI 0.77 - 0.94) and dominant models (REM OR 0.82, 95 % CI 0.77 - 0.87; Table 1 and Figure 3). In the same way, the rs34536443 C allele was associated with protection for autoimmune diseases under allele contrast (REM OR 0.68, 95 % CI 0.61 - 0.76) and dominant (REM OR 0.75, 95 % CI 0.58 - 0.98) models (Table 1 and Figure 4).

**Figure 5 -:**
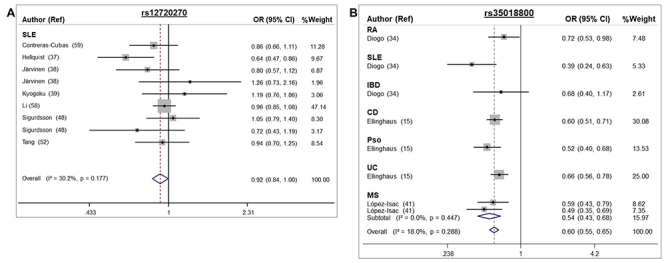
Forest plots showing individual and pooled OR (95 % CI) for the associations between the *TYK2* rs12720270 (A) and rs35018800 SNPs (B) and autoimmune diseases, under an allele contrast model.

The A allele of the rs12720270 SNP conferred protection for SLE under the allele contrast model [FEM OR 0.92, 95 % CI 0.84 - 1.00 (P = 0.041); Table 1 and Figure 5]. Moreover, after stratification by ethnicity, the A allele was associated with protection for SLE in Caucasians (FEM OR 0.84, 95 % CI 0.72-0.98) but not in Asians (Table 1). Besides SLE, this SNP was not evaluated in other autoimmune diseases. The A allele of the rs35018800 SNP also conferred protection for autoimmune diseases (FEM OR 0.60, 95 % CI 0.55 - 0.65, allele contrast model; Table 1 and Figure 5) in European populations.

### Sensitivity analyses and publication bias

When significant inter-study heterogeneities were observed, sensitivity analyses were carried out in order to estimate the influence of each individual study on the meta-analysis results obtained when assuming the allele contrast model. This was performed by repeating meta-analyses excluding a different study each time. Our results showed two studies (International Consortium for Systemic Lupus Erythematosus Genetics *et al.*, 2008; Tang et al., 2015) explained the observed heterogeneity in the meta-analysis of the rs280500 SNP since their exclusion significantly decreased the heterogeneity (all studies: I² = 42.6 %, P = 0.138, and after exclusion: I² 0.0 %, P = 0.901). However, the exclusion of these two studies from the rs280500 meta-analysis did not change the lack of the association of this SNP with autoimmune diseases. Moreover, the exclusion of one study (Genetic Analysis of Psoriasis et al., 2010) from the rs280519 meta-analysis decreased the observed heterogeneity (I² = 27.7 %, P = 0.165). Importantly, the exclusion of this study significantly changed the pooled OR for this SNP, which was now associated with risk for autoimmune diseases (OR 1.11, 95 % CI 1.05 - 1.18). Of note, meta-analyses of the rs2304256, rs12720356, and rs34536443 SNPs still presented significant heterogeneity after sensitivity analyses.

Funnel plots and Egger’s tests were performed to investigate the presence of possible publication bias in those meta-analyses containing at least 10 studies, which were those performed for the rs12720356, rs2304256, rs280519, and rs34536443 SNPs (Figure S3A-D). No significant publication bias was observed for the rs2304256, rs280519, and rs34536443 SNPs. However, funnel plot and Egger’s test indicated a significant publication bias in the rs12720356 meta-analysis (P = 0.037; Figure S3A). Trim and Fill analysis was performed to account for this bias, and the results indicated that the pooled OR obtained for this SNP did not change significantly since the adjusted effect was similar to the original effect.

## Discussion

To further investigate the possible effects of the *TYK2* SNPs on susceptibility for autoimmune diseases, we performed meta-analyses of 34 published articles on the field. Our results suggest the minor alleles of rs2304256, rs12720270, rs12720356, rs34536443, and rs35018800 SNPs are associated with protection against autoimmune diseases, while the rs280519A allele is associated with risk for SLE. The rs280496, rs280500, and rs280523 SNPs do not seem to be associated with autoimmune diseases in the investigated populations.

Our meta-analysis for the rs280519 SNP included 14 studies (13 969 cases and 29 167 controls) and showed the A allele of this SNP is associated with risk for SLE. This SNP does not seem to be associated with CD, UC, and Pso. In contrast, two previous meta-analyses did not show any association of this SNP with autoimmune diseases (Tao et al., 2011; Yin et al., 2018). The discordant results may be due to the small number of studies included in the meta-analyses by Tao *et al*. ( 2011), which included 3 studies with CD and SLE, and by Yin *et al*. (2018), which included 4 studies with SLE. The A allele of this SNP does not cause an amino acid substitution, but it is located in a splice site of the *TYK2* gene (Lopez-Rodriguez et al., 2017). No study has evaluated if this SNP has an impact on TYK2 function. Linkage disequilibrium (LD) analyses suggest the rs280519 SNP is on the same haplotype block of the rs2304256 and rs12720270 SNPs (Kyogoku et al., 2009); thus, the rs280519 SNP could be a marker of the functional rs2304256 SNP.

Our meta-analysis for the rs2304256 SNP included 25 studies (23 827 cases and 35 760 controls) and showed the A allele of this SNP is associated with protection against autoimmune diseases (SLE, CD, UC, T1DM, MS, and RA) in all inheritance models analyzed. This association was confirmed in SLE and MS diseases, although the lack of individual associations with CD, UC, T1DM, and RA might be due to the small number of studies/sample sizes for each disease. In addition, after stratification by ethnicity, this SNP remained associated with autoimmune diseases in Caucasians (REM OR 0.89, 95 % CI 0.81 - 0.98, allele contrast model) but not in Asians or populations of mixed ethnicity (from Southern Brazil), which can be attributed to the fact that the studies in Asian and Brazilian populations presented a small number of subjects. Our results regarding this SNP are in agreement with the results of two previous meta-analyses (Tao et al., 2011; Lee and Bae, 2016). The meta-analysis performed by Tao *et al*. (2011) included only 11 studies and showed the rs2304256 A allele conferred protection for SLE, RA, UC, and CD (OR 0.78, 95 % CI 0.70 - 0.87, for the allele contrast model). In 2016, Lee and Bae (2016) published a meta-analysis of 12 studies, showing the rs2304256 A allele was associated with protection against SLE and RA in Caucasians (OR 0.82, 95 % CI 0.70 - 0.89) but not in Asians (Lee and Bae, 2016). In addition, Zuvich et al. (2010) demonstrated the rs2304256 A allele was associated with protection for MS (OR 0.90) in North American and British subjects. This study was not included in our meta-analysis due to lack of required data.

The A allele of the rs2304256 causes a substitution of valine to phenylalanine at position 362 in the JAK-homology 4 (JH4) region, which is a crucial domain for interaction of TYK2 with IFNAR1 and its function, maintaining the expression of *IFNAR1* on cell membranes (Tao et al., 2011; Marroqui et al., 2015). Li et al. (2020) showed the rs2304256 A allele affects the *TYK2* pre-mRNA processing since it destroys a putative exonic splicing enhancer; thus, promoting the inclusion of exon 8 in the mRNA, which is essential for TYK2 binding to cytokine receptors. Marroqui *et al*. (2015) isolated B lymphoblastoid cell lines (BLCLs) from subjects carrying the rs2304256 A/A genotype and demonstrated less marked IFN-α-induced STAT1 phosphorylation compared with subjects carrying the C/C genotype (3.5-fold STAT1 phosphorylation *vs*. 5.7-fold increase). Interestingly, TYK2 inhibition decreased cytokine-induced apoptosis and pro-inflammatory pathways in pancreatic beta-cells via inhibition of the IFN-I signaling and consequent decrease in STAT1/2 phosphorylation (Marroqui *et al.*, 2015). It is well known that the initial attack in autoimmune diseases is usually followed by an inflammatory response caused by autoreactive cytotoxic cells, which then activates the release of pro-inflammatory cytokines and apoptosis via JAK-STAT pathways (Stuart and Hughes, 2002; Coomans de Brachene et al., 2020). Thus, taken together, these studies suggest the rs2304256 SNP decreases TYK2 activity and, consequently, the inflammatory response and apoptosis, explaining its association with protection against autoimmune diseases.

Our meta-analysis for the rs12720356 SNP included 20 studies (69 788 cases and 177 437 controls) and showed the C allele of this SNP provides protection for autoimmune diseases (SLE, RA, IBD, CD, Pso, UC, and MS) under allele contrast and dominant models. Among the 20 studies that evaluated this SNP, 18 were performed in Caucasian subjects. Accordingly, the meta-analysis conducted by Lee and Bae (2016) included 6 studies with rheumatic diseases and showed a similar result to ours (OR 0.81, 95 % CI 0.66 - 0.99). In contrast, another small meta-analysis, which included 4 035 cases with SLE, RA, or CD and 2 953 controls, was not able to find any association between the rs12720356 SNP and these diseases, possible because of the small number of evaluated studies (n = 4) and sample sizes (Tao et al., 2011). The C allele of the rs12720356 SNP leads to a isoleucine to serine substitution at position 684 in the pseudo-kinase region JAK-homology 2 (JH2) of *TYK2*. This region is required for the binding of IFN-I to IFNAR1 (Sigurdsson et al., 2005). Enerbäck *et al*. (2018) analyzed peripheral blood mononuclear cells (PBMCs) from patients with Pso carrying the A allele (n = 10) *vs*. patients with the C allele (n = 10) of this SNP. PBMCs from subjects carrying the C allele showed reduced phosphorylated (p)-STAT4 levels after induction with IL-12 compared to the A/A genotype, suggesting the rs12720356 C allele may have a functional impact on TYK2 function and, consequently, immunity (Enerback et al., 2018).

Our meta-analysis for the rs34536443 SNP included 19 studies (50 011 cases and 95 923 controls) and showed the C allele is associated with protection against autoimmune diseases (MS, RA, SLE, IBD, Pso, and T1DM) under both allele contrast and dominant models. This association was confirmed for SLE and MS; however, for IBD, Pso, and T1DM, we had a small number of studies to individually conclude about the associations with these diseases. Of note, most of the studies were performed in Caucasian subjects. Moreover, two studies (Johnson et al., 2010; International Multiple Sclerosis Genetics et al., 2013) were not included in our meta-analysis due to lack of data. Johnson *et al*. (2010) demonstrated the rs34536443 C allele was associated with risk for MS (OR 2.04, 95 % CI 1.01 - 4.08) in African-Americans (Johnson *et al.*, 2010). In contrast, another study including 14 498 patients with MS and 24 091 controls of European ancestry suggested this allele conferred protection for MS (OR 0.95; P = 1.2x10^-8^), which is in accordance to our results (International Multiple Sclerosis Genetics *et al.*, 2013). Tao et al. (2011) also performed a meta-analysis of the rs34536443, including 9 studies with MS (10 642 MS patients / 10 620 controls), and showed the C allele was associated with protection against this disease (OR 0.76, 95 % CI 0.69 - 0.84) (Tao *et al.*, 2011).

The rs34536443 SNP is located in exon 21 and causes a change of a proline to alanine at position 1104 within the kinase domain of TYK2 (Peluso et al., 2013; Gorman et al., 2019). The C allele of this SNP seems to be functional since it decreased the IFN-α induced-pSTAT1 levels in PBMCs compared to cells obtained from patients carrying the G allele, thus reducing IFNAR signaling (Gorman *et al.*, 2019). The rs34536443 C allele also decreased IL-23 and IL-12 induced-p-STAT3 levels in a murine model of MS (Gorman *et al.*, 2019). Accordingly, PBMCs of patients with MS carrying the C allele of this SNP also showed reduced IFNβ induced-p-STAT2 levels compared to patients with the G allele (Couturier et al., 2011).

Our meta-analysis for the rs12720270 SNP included 9 studies (2 792 cases and 5 184 controls) and showed the A allele of this SNP was associated with protection against SLE. In contrast, 3 previous meta-analyses (Tao et al., 2011; Lee and Bae, 2016; Yin et al., 2018), including only 3 to 5 studies with SLE patients, were not able to show any association between this SNP and SLE. This SNP is located in intron 7 of *TYK2* gene, most specifically 36 nt upstream of the intron 7/exon 8 boundary (Li et al., 2020). The rs12720270 SNP is in strong LD with the functional rs2304256 and the rs280519 SNPs (Contreras-Cubas et al., 2019; Li *et al.*, 2020). However, it also seems to be functional since *in silico* analysis and cell line experiments suggested the A allele breaks a splicing-branch point in the intron, promoting the inclusion of exon 8 in the mature *TYK2* mRNA; thus, influencing TYK2 activity (Li *et al.*, 2020).

Our meta-analysis for the rs35018800 SNP included 8 studies (61 241 cases and 163 386 controls) and demonstrated the A allele of this SNP is associated with protection against autoimmune diseases (RA, SLE, IBD, CD, Pso, UC, and MS) in Caucasian subjects. No study has evaluated this SNP in other ethnicities. We were not able to determine if this SNP provides differential protection for a given autoimmune disease since we had a small number of studies for each disease. The rs35018800 SNP causes a substitution of an alanine to valine at position 928 within the kinase domain of TYK2 (Lopez-Isac et al., 2016). To date, there is no available information if this SNP has a functional significance. 

Autoimmune diseases share common etiological pathways and, as a result, they may also share some similar genetic factors (Gutierrez-Roelens and Lauwerys, 2008; Luan et al., 2017). Indeed, our present meta-analysis confirms that the rs2304256, rs12720270, rs12720356, rs34536443, rs35018800, and rs280519 SNPs influence the susceptibility to different autoimmune diseases. However, the size of the effect of each individual SNP on a specific autoimmune disease might be affected by the interaction with other environmental and genetic factors involved in that disease. Thus, additional studies with larger sample sizes are required in order to clarify the effects of the rs2304256, rs12720270, rs12720356, rs34536443, rs35018800, and rs280519 SNPs on each autoimmune disease analyzed here. Moreover, since some of the analyzed SNPs (rs12720270, rs2304256, and rs280519) are in strong LD, future functional studies should evaluate which is(are) the functional(s) SNP(s) in a LD block or if they are interacting in the susceptibility for the autoimmune diseases. 

Despite all the efforts, the results of the present meta-analysis should be interpreted within the context of few limitations. First, we tried to retrieve all published articles, but we cannot exclude the possibility that small negative studies could have been lost. Although we did not observe publication bias for the rs2304256, rs280519, and rs34536443 SNPs, a significant publication bias was present in the meta-analysis of the rs12720356 SNP. However, Trim and Fill analysis demonstrated that the adjusted effect did not change significantly, indicating that the number of missing studies needed to reverse the bias is smaller than the number of missing studies needed to nullify the effect (Brondani et al., 2014). Second, we only analyzed those articles written in English, Spanish or Portuguese; hence, we could have lost few articles written in other languages. Third, we were not able to perform meta-regression analyses to explain the observed heterogeneity because of lack of data regarding age and gender. Despite of that, we performed sensibility analysis for those SNPs that showed significant heterogeneity in the respective meta-analyses. The exclusion of two studies (International Consortium for Systemic Lupus Erythematosus Genetics *et al.*, 2008; Tang et al., 2015) from the rs280500 meta-analysis decreased its heterogeneity, but did not change the observed result. However, the exclusion of one study (Genetic Analysis of Psoriasis et al., 2010), with a large sample size, explained the heterogeneity detected in the rs280519 meta-analysis. After exclusion of this study, the rs280519 SNP was associated with risk for autoimmune diseases, suggesting that heterogeneity among studies might have influenced the results. Fourth, as already mentioned, we could not include 3 articles (Johnson et al., 2010; Zuvich et al., 2010; International Multiple Sclerosis Genetics et al., 2013) in our meta-analyses due to the lack of data. Fifth, the rs280496, rs280523, and rs35018800 SNPs were investigated by few studies, thus we were not able to stratify their meta-analyses by disease type. In addition, ethnic-specific association studies are required to confirm genetic associations in different populations (Lee et al., 2012), since we could not identify differences among ethnicities in the meta-analyses of the rs34536443, rs280500, rs280496, rs12720356, and rs35018800 SNPs due to the small number of studies evaluating different ethnicities.

In conclusion, our results suggest that the minor alleles of the rs2304256, rs12720270, rs12720356, rs34536443, and rs35018800 SNPs are involved in the protection against autoimmune diseases, and the A allele of the rs280519 SNP is associated with risk for SLE. In addition, our results indicate that the rs280496, rs280500, and rs280523 SNPs are not associated with autoimmune diseases. Additional studies with larger sample sizes are necessary to clarify the impact of each *TYK2* SNP on susceptibility for different autoimmune diseases. Functional studies are also needed to elucidate which are the *TYK2* SNPs with the highest impact on *TYK2* function and, consequently, on autoimmune diseases.

## Supplementary material

The following online material is available for this article:

Figure S1 - Forest plots showing individual and pooled OR (95 % CI) for the associations between the *TYK2* rs280496 and rs280500 SNPs and autoimmune diseases, under an allele contrast model.

Figure S2 - Forest plots showing individual and pooled OR (95 % CI) for the associations between the *TYK2* rs280523 and rs280519 SNPs and autoimmune diseases, under an allele contrast model.

Figure S3 - Begg’s funnel plots for publication bias test for *TYK2* SNPs: A) rs12720356, B) rs2304256, C) rs280519, and D) rs34536443.

Table S1 - Genotype and allele distributions of *TYK2* rs280496, rs280500, rs280519, rs280523, rs2304256, rs12720270, rs12720356, rs34536443, and rs35018800 SNPs in patients with autoimmune diseases and control subjects.

Table S2 - Newcastle-Ottawa quality assessment scale for the studies included in the meta-analyses.

## References

[B1] Almlof JC, Alexsson A, Imgenberg-Kreuz J, Sylwan L, Backlin C, Leonard D, Nordmark G, Tandre K, Eloranta ML, Padyukov L (2017). Novel risk genes for systemic lupus erythematosus predicted by
random forest classification. Sci Rep.

[B2] Alonso-Perez E, Suarez-Gestal M, Calaza M, Blanco FJ, Suarez A, Santos MJ, Papasteriades C, Carreira P, Pullmann R, Ordi-Ros J (2014). Lack of replication of higher genetic risk load in men than in
women with systemic lupus erythematosus. Arthritis Res Ther.

[B3] Ban M, Goris A, Lorentzen AR, Baker A, Mihalova T, Ingram G, Booth DR, Heard RN, Stewart GJ, Bogaert E (2009). Replication analysis identifies TYK2 as a multiple sclerosis
susceptibility factor. Eur J Hum Genet.

[B4] Brondani LA, Souza BM, Assmann TS, Bouças AP, Bauer AC, Canani LH, Crispim D (2014). Association of the UCP polymorphisms with susceptibility to
obesity: case-control study and meta-analysis. Mol Biol Rep.

[B5] Can G, Tezel A, Gurkan H, Can H, Yilmaz B, Unsal G, Soylu AR, Umit HC (2015). Tyrosine kinase-2 gene polymorphisms are associated with
ulcerative colitis and Crohn’s disease in Turkish Population. Clin Res Hepatol Gastroenterol.

[B6] Clark MF, Baudouin SV (2006). A systematic review of the quality of genetic association studies
in human sepsis. Intensive Care Med.

[B7] Contreras-Cubas C, Garcia-Ortiz H, Velazquez-Cruz R, Barajas-Olmos F, Baca P, Martinez-Hernandez A, Barbosa-Cobos RE, Ramirez-Bello J, Lopez-Hernandez MA, Svyryd Y (2019). Catalytically impaired TYK2 variants are protective against
childhood- and adult-onset systemic lupus erythematosus in
Mexicans. Sci Rep.

[B8] Coomans de Brachene A, Castela A, Op de Beeck A, Mirmira RG, Marselli L, Marchetti P, Masse C, Miao W, Leit S, Evans-Molina C (2020). Preclinical evaluation of tyrosine kinase 2 inhibitors for human
beta-cell protection in type 1 diabetes. Diabetes Obes Metab.

[B9] Couturier N, Bucciarelli F, Nurtdinov RN, Debouverie M, Lebrun-Frenay C, Defer G, Moreau T, Confavreux C, Vukusic S, Cournu-Rebeix I (2011). Tyrosine kinase 2 variant influences T lymphocyte polarization
and multiple sclerosis susceptibility. Brain.

[B10] Deng YN, Bellanti JA, Zheng SG (2019). Essential kinases and transcriptional regulators and their roles
in autoimmunity. Biomolecules.

[B11] Diogo D, Bastarache L, Liao KP, Graham RR, Fulton RS, Greenberg JD, Eyre S, Bowes J, Cui J, Lee A (2015). TYK2 protein-coding variants protect against rheumatoid arthritis
and autoimmunity, with no evidence of major pleiotropic effects on
non-autoimmune complex traits. PLoS One.

[B12] Duval S, Tweedie R (2000). Trim and fill: A simple funnel-plot-based method of testing and
adjusting for publication Bias in meta-analysis. Biometrics.

[B13] Egger M, Davey Smith G, Schneider M, Minder C (1997). Bias in meta-analysis detected by a simple, graphical
test. BMJ.

[B14] Ellinghaus D, Jostins L, Spain SL, Cortes A, Bethune J, Han B, Park YR, Raychaudhuri S, Pouget JG, Hubenthal M (2016). Analysis of five chronic inflammatory diseases identifies 27 new
associations and highlights disease-specific patterns at shared
loci. Nat Genet.

[B15] Enerback C, Sandin C, Lambert S, Zawistowski M, Stuart PE, Verma D, Tsoi LC, Nair RP, Johnston A, Elder JT (2018). The psoriasis-protective TYK2 I684S variant impairs IL-12
stimulated pSTAT4 response in skin-homing CD4+ and CD8+ memory
T-cells. Sci Rep.

[B16] Genetic Analysis of Psoriasis C, the Wellcome Trust Case Control C, Strange A, Capon F, Spencer CC, Knight J, Weale ME, Allen MH, Barton A, Band G (2010). A genome-wide association study identifies new psoriasis
susceptibility loci and an interaction between HLA-C and
ERAP1. Nat Genet.

[B17] Ghoreschi K, Laurence A, O’Shea JJ (2009). Janus kinases in immune cell signaling. Immunol Rev.

[B18] Gorman JA, Hundhausen C, Kinsman M, Arkatkar T, Allenspach EJ, Clough C, West SE, Thomas K, Eken A, Khim S (2019). The TYK2-P1104A autoimmune protective variant limits coordinate
signals required to generate specialized T cell subsets. Front Immunol.

[B19] Graciolo V, Welter M, Campos LP, Martins BR, Souza SW, França SN, Réa RR, Picheth G, Rego FGM (2019). Polymorphism V362F (rs2304256) of tyrosine kinase 2 is not
associated with childhood- or adulthood-onset type 1 diabetes in southern
Brazil. Genet Mol Res.

[B20] Graham DSC, Morris DL, Bhangale TR, Criswell LA, Syvanen AC, Ronnblom L, Behrens TW, Graham RR, Vyse TJ (2011). Association of NCF2, IKZF1, IRF8, IFIH1, and TYK2 with systemic
lupus erythematosus. PLoS Genet.

[B21] Gutierrez-Roelens I, Lauwerys BR (2008). Genetic susceptibility to autoimmune disorders: Clues from gene
association and gene expression studies. Curr Mol Med.

[B22] Hellquist A, Jarvinen TM, Koskenmies S, Zucchelli M, Orsmark-Pietras C, Berglind L, Panelius J, Hasan T, Julkunen H, D’Amato M (2009). Evidence for genetic association and interaction between the TYK2
and IRF5 genes in systemic lupus erythematosus. J Rheumatol.

[B23] Higgins JP, Thompson SG, Deeks JJ, Altman DG (2003). Measuring inconsistency in meta-analyses. BMJ.

[B24] Harley JB, Alarcón-Riquelme ME, Criswell LA, Jacob CO, Kimberly RP, Moser KL, Tsao BP, Vyse TJ, International Consortium for Systemic Lupus Erythematosus
Genetics (2008). Genome-wide association scan in women with systemic lupus
erythematosus identifies susceptibility variants in ITGAM, PXK, KIAA1542 and
other loci. Nat Genet.

[B25] Beecham AH, Patsopoulos NA, Xifara DK, Davis MF, Kemppinen A, Cotsapas C, Shah TS, Spencer C, Booth D, International Multiple Sclerosis Genetics C (2013). Analysis of immune-related loci identifies 48 new susceptibility
variants for multiple sclerosis. Nat Genet.

[B26] Jarvinen TM, Hellquist A, Koskenmies S, Einarsdottir E, Koskinen LL, Jeskanen L, Berglind L, Panelius J, Hasan T, Ranki A (2010). Tyrosine kinase 2 and interferon regulatory factor 5
polymorphisms are associated with discoid and subacute cutaneous lupus
erythematosus. Exp Dermatol.

[B27] Johnson BA, Wang J, Taylor EM, Caillier SJ, Herbert J, Khan OA, Cross AH, De Jager PL, Gourraud PA, Cree BC (2010). Multiple sclerosis susceptibility alleles in African
Americans. Genes Immun.

[B28] Kyogoku C, Morinobu A, Nishimura K, Sugiyama D, Hashimoto H, Tokano Y, Mimori T, Terao C, Matsuda F, Kuno T (2009). Lack of association between tyrosine kinase 2 (TYK2) gene
polymorphisms and susceptibility to SLE in a Japanese
population. Mod Rheumatol.

[B29] Langefeld CD, Ainsworth HC, Cunninghame Graham DS, Kelly JA, Comeau ME, Marion MC, Howard TD, Ramos PS, Croker JA, Morris DL (2017). Transancestral mapping and genetic load in systemic lupus
erythematosus. Nat Commun.

[B30] Lee YH, Bae SC (2016). Association between TYK2 polymorphisms and susceptibility to
autoimmune rheumatic diseases: a meta-analysis. Lupus.

[B31] Lee YH, Choi SJ, Ji JD, Song GG (2012). Associations between PXK and TYK2 polymorphisms and systemic
lupus erythematosus: a meta-analysis. Inflamm Res.

[B32] Lees CW, Barrett JC, Parkes M, Satsangi J (2011). New IBD genetics: common pathways with other
diseases. Gut.

[B33] Li P, Chang YK, Shek KW, Lau YL (2011). Lack of association of TYK2 gene polymorphisms in Chinese
patients with systemic lupus erythematosus. J Rheumatol.

[B34] Li Z, Rotival M, Patin E, Michel F, Pellegrini S (2020). Two common disease-associated TYK2 variants impact exon splicing
and TYK2 dosage. PLoS One.

[B35] Lian LH, Lau TP, Lee VL, Lee WS, Hilmi I, Goh KL, Chua KH (2013). Lack of association between TYK2 and STAT3 genes and Crohn’s
disease in the Malaysian population. Genet Mol Res.

[B36] Lopez-Isac E, Campillo-Davo D, Bossini-Castillo L, Guerra SG, Assassi S, Simeon CP, Carreira P, Ortego-Centeno N, Garcia de la Pena P, Spanish Scleroderma Group (2016). Influence of TYK2 in systemic sclerosis susceptibility: a new
locus in the IL-12 pathway. Ann Rheum Dis.

[B37] Lopez-Rodriguez R, Hernandez-Bartolome A, Borque MJ, Rodriguez-Munoz Y, Martin-Vilchez S, Garcia-Buey L, Gonzalez-Moreno L, Real-Martinez Y, Munoz de Rueda P, Salmeron J (2017). Interferon-related genetic markers of necroinflammatory activity
in chronic hepatitis C. PLoS One.

[B38] Luan M, Shang Z, Teng Y, Chen X, Zhang M, Lv H, Zhang R (2017). The shared and specific mechanism of four autoimmune
diseases. Oncotarget.

[B39] Marrack P, Kappler J, Kotzin BL (2001). Autoimmune disease: why and where it occurs. Nat Med.

[B40] Marroqui L, Santos RS, Fløyel T, Grieco FA, Santin I, de Beeck AO, Marselli L, Marchetti P, Pociot F, Eizirik DL (2015). TYK2, a candidate gene for Type 1 Diabetes, modulates apoptosis
and the innate immune response in human pancreatic b-cells. Diabetes.

[B41] Melsen WG, Bootsma MC, Rovers MM, Bonten MJ (2014). The effects of clinical and statistical heterogeneity on the
predictive values of results from meta-analyses. Clin Microbiol Infect.

[B42] Mero IL, Lorentzen AR, Ban M, Smestad C, Celius EG, Aarseth JH, Myhr KM, Link J, Hillert J, Olsson T (2010). A rare variant of the TYK2 gene is confirmed to be associated
with multiple sclerosis. Eur J Hum Genet.

[B43] Mohamadhosseini A, Mansouri R, Javinani A, Ganjouei AA, Akhlaghi M, Aslani S, Hamzeh E, Jamshidi A, Ahmadzadeh N, Mahmoudi M (2019). Single nucleotide polymorphism of TYK2 gene and susceptibility to
rheumatoid arthritis in Iranian population. Avicenna J Med Biotechnol.

[B44] Moher D, Liberati A, Tetzlaff J, Altman DG (2009). Preferred reporting items for systematic reviews and
meta-analyses: The PRISMA statement. PLoS Medicine.

[B45] Myrthianou E, Zervou MI, Budu-Aggrey A, Eliopoulos E, Kardassis D, Boumpas DT, Kougkas N, Barton A, Sidiropoulos P, Goulielmos GN (2017). Investigation of the genetic overlap between rheumatoid arthritis
and psoriatic arthritis in a Greek population. Scand J Rheumatol.

[B46] Nagafuchi S, Kamada-Hibio Y, Hirakawa K, Tsutsu N, Minami M, Okada A, Kai K, Teshima M, Moroishi A, Murakami Y (2015). TYK2 promoter variant and Diabetes Mellitus in the
Japanese. EBioMedicine.

[B47] O’Shea JJ, Plenge R (2012). JAK and STAT signaling molecules in immunoregulation and
immune-mediated disease. Immunity.

[B48] Odhams CA, Cunninghame Graham DS, Vyse TJ (2017). Profiling RNA-Seq at multiple resolutions markedly increases the
number of causal eQTLs in autoimmune disease. PLoS Genet.

[B49] Peluso C, Christofolini DM, Goldman CS, Mafra FA, Cavalcanti V, Barbosa CP, Bianco B (2013). TYK2 rs34536443 polymorphism is associated with a decreased
susceptibility to endometriosis-related infertility. Hum Immunol.

[B50] Prieto-Perez R, Solano-Lopez G, Cabaleiro T, Roman M, Ochoa D, Talegon M, Baniandres O, Lopez-Estebaranz JL, de la Cueva P, Dauden E (2015). Polymorphisms associated with age at onset in patients with
Moderate-to-Severe Plaque Psoriasis. J Immunol Res.

[B51] Qiu W, Pham K, James I, Nolan D, Castley A, Christiansen FT, Czarniak P, Luo Y, Wu J, Garlepp M (2013). The influence of non-HLA gene polymorphisms and interactions on
disease risk in a Western Australian multiple sclerosis
cohort. J Neuroimmunol.

[B52] Rose NR (2016). Prediction and prevention of autoimmune disease in the 21st
Century: A review and preview. Am J Epidemiol.

[B53] Sato K, Shiota M, Fukuda S, Iwamoto E, Machida H, Inamine T, Kondo S, Yanagihara K, Isomoto H, Mizuta Y (2009). Strong evidence of a combination polymorphism of the tyrosine
kinase 2 gene and the signal transducer and activator of transcription 3
gene as a DNA-based biomarker for susceptibility to Crohn’s disease in the
Japanese population. J Clin Immunol.

[B54] Shaiq PA, Stuart PE, Latif A, Schmotzer C, Kazmi AH, Khan MS, Azam M, Tejasvi T, Voorhees JJ, Raja GK (2013). Genetic associations of psoriasis in a Pakistani
population. Br J Dermatol.

[B55] Sigurdsson S, Nordmark G, Goring HHH, Lindroos K, Wiman AC, Sturfelt G, Jonsen A, Dahlqvist SR, Moller B, Kere J (2005). Polymorphisms in the tyrosine kinase 2 and interferon regulatory
factor 5 genes are associated with systemic lupus
erythematosus. Am J Hum Genet.

[B56] Sigurdsson S, Padyukov L, Kurreeman FA, Liljedahl U, Wiman AC, Alfredsson L, Toes R, Ronnelid J, Klareskog L, Huizinga TW (2007). Association of a haplotype in the promoter region of the
interferon regulatory factor 5 gene with rheumatoid
arthritis. Arthritis Rheum.

[B57] Souza BM, Brondani LA, Bouças AP, Sortica DA, Kramer CK, Canani LH, Leitão CB, Crispim D (2013). Associations between UCP1 -3826A/G, UCP2 -866G/A, Ala55Val and
Ins/Del, and UCP3 -55C/T Polymorphisms and susceptibility to type 2 diabetes
mellitus: Case-control study and meta-analysis. PloS One.

[B58] Strobl B, Stoiber D, Sexl V (2011). Tyrosine kinase 2 (TYK2) in cytokine signalling and host
immunity. Front Biosci.

[B59] Stroup DF, Berlin JA, Morton SC, Olkin I, Williamson GD, Rennie D, Moher D, Becker BJ, Sipe TA, Thacker SB (2000). Meta-analysis of observational studies in epidemiology: A
proposal for reporting. JAMA.

[B60] Stuart L, Hughes J (2002). Apoptosis and autoimmunity. Nephrol Dial Transplant.

[B61] Suarez-Gestal M, Calaza M, Dieguez-Gonzalez R, Perez-Pampin E, Pablos JL, Navarro F, Narvaez J, Marenco JL, Herrero-Beaumont G, Fernandez-Gutierrez B (2009). Rheumatoid arthritis does not share most of the newly identified
systemic lupus erythematosus genetic factors. Arthritis Rheum.

[B62] Suarez-Gestal M, Calaza M, Endreffy E, Pullmann R, Ordi-Ros J, Sebastiani GD, Ruzickova S, Jose Santos M, Papasteriades C, Marchini M (2009). Replication of recently identified systemic lupus erythematosus
genetic associations: a case-control study. Arthritis Res Ther.

[B63] Tang L, Wan P, Wang Y, Pan J, Wang Y, Chen B (2015). Genetic association and interaction between the IRF5 and TYK2
genes and systemic lupus erythematosus in the Han Chinese
population. Inflamm Res.

[B64] Tao JH, Zou YF, Feng XL, Li J, Wang F, Pan FM, Ye DQ (2011). Meta-analysis of TYK2 gene polymorphisms association with
susceptibility to autoimmune and inflammatory diseases. Mol Biol Rep.

[B65] Wells GA, Shea B, O’Connell D, Peterson J, Welch V, Losos M, Tugwell P (2020). The Newcastle-Ottawa Scale (NOS) for assessing the quality of
nonrandomised studies in meta-analyses.

[B66] Westra HJ, Martinez-Bonet M, Onengut-Gumuscu S, Lee A, Luo Y, Teslovich N, Worthington J, Martin J, Huizinga T, Klareskog L (2018). Fine-mapping and functional studies highlight potential causal
variants for rheumatoid arthritis and type 1 diabetes. Nat Genet.

[B67] Yamaoka K, Saharinen P, Pesu M, 3rd Holt VE, Silvennoinen O, O’Shea JJ (2004). The Janus kinases (Jaks). Genome Biol.

[B68] Yin Q, Wu LC, Zheng L, Han MY, Hu LY, Zhao PP, Bai WY, Zhu XW, Xia JW, Wang XB (2018). Comprehensive assessment of the association between genes on
JAK-STAT pathway (IFIH1, TYK2, IL-10) and systemic lupus erythematosus: a
meta-analysis. Arch Dermatol Res.

[B69] Zaplakhova OV, Nasibullin TR, Tuktarova IA, Timasheva YR, Erdman VV, Bakhtiyarova KZ, Mustafina OE (2018). Associations of polymorphic DNA markers and their combinations
with multiple sclerosis. Russ J Genet.

[B70] Zintzaras E, Lau J (2008). Synthesis of genetic association studies for pertinent
gene-disease associations requires appropriate methodological and
statistical approaches. J Clin Epidemiol.

[B71] Zuvich RL, McCauley JL, Oksenberg JR, Sawcer SJ, De Jager PL, Aubin C, Cross AH, Piccio L, Aggarwal NT, International Multiple Sclerosis Genetics C (2010). Genetic variation in the IL7RA/IL7 pathway increases multiple
sclerosis susceptibility. Hum Genet.

