# Supplementary material for: Association of TYK2 polymorphisms with autoimmune diseases: A comprehensive and updated systematic review with meta-analysis
Source: Genet Mol Biol. 2021 May 3;44(2):e20200425. doi: 10.1590/1678-4685-GMB-2020-0425 (PMC8097517; doi:10.1590/1678-4685-GMB-2020-0425)
Supplement: Figure S2 - [file 1415-4757-GMB-44-2-e20200425-s2.pdf]

Genetics and Molecular Biology
https://doi.org/10.1590/1678-4685-GMB-2020-0425

Supplementary Material to "Association of *TYK2* polymorphisms with autoimmune diseases: A comprehensive and updated systematic review with meta-analysis"

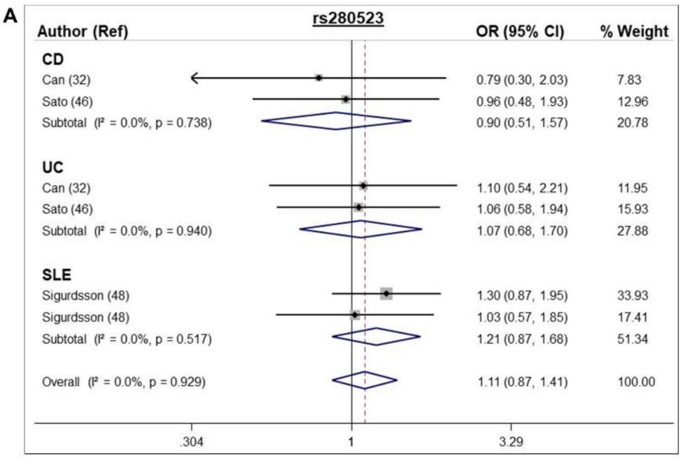

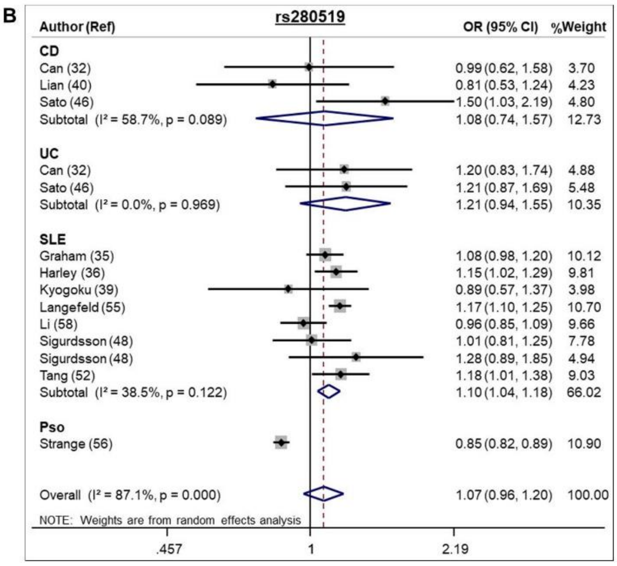

Figure S2 - Forest plots showing individual and pooled OR (95% CI) for the associations between the *TYK2* rs280523 and rs280519 SNPs and autoimmune diseases, under an allele contrast model.
