# Supplementary material for: Association of TYK2 polymorphisms with autoimmune diseases: A comprehensive and updated systematic review with meta-analysis
Source: Genet Mol Biol. 2021 May 3;44(2):e20200425. doi: 10.1590/1678-4685-GMB-2020-0425 (PMC8097517; doi:10.1590/1678-4685-GMB-2020-0425)
Supplement: Table S1 - [file 1415-4757-GMB-44-2-e20200425-s4.pdf]

Genetics and Molecular Biology
https://doi.org/10.1590/1678-4685-GMB-2020-0425

Supplementary Material to "Association of *TYK2* polymorphisms with autoimmune diseases: A comprehensive and updated systematic review with meta-analysis"

Table S1 - Genotype and allele distributions of the *TYK2* rs280496, rs280500, rs280519, rs280523, rs2304256, rs12720270, rs12720356, rs34536443, and rs35018800 SNPs in patients with autoimmune diseases (cases) and controls.

rs280496			**Cases (n) by total and genotype**				**Controls (n) by total and genotype**				**G allele frequency (%)**	
**Author, year**	**Disease**	**Ethnicity**	**Total**	**C/C**	**C/G**	**G/G**	**Total**	**C/C**	**C/G**	**G/G**	**Cases**	**Controls**
Can[a], 2015	CD	Asian	60	34	24	2	89	51	34	4	0.233	0.236
Can[b], 2015	UC	Asian	151	82	60	9	89	51	34	4	0.258	0.236
Sato[a], 2009	CD	Asian	83	61	22	0	200	162	38	0	0.133	0.095
Sato[b], 2009	UC	Asian	114	ND	ND	ND	200	162	38	0	0.103	0.095

rs280500			**Cases (n) by total and genotype**				**Controls (n) by total and genotype**				**A allele frequency (%)**	
**Author, year**	**Disease**	**Ethnicity**	**Total**	**G/G**	**G/A**	**A/A**	**Total**	**G/G**	**G/A**	**A/A**	**Cases**	**Controls**
Contreras-Cubas, 2019	SLE	Mixed	368	ND	ND	ND	516	ND	ND	ND	0.061	0.052
Harley, 2008	SLE	Caucasian	720	ND	ND	ND	2,337	ND	ND	ND	0.160	0.160

Li, 2011	SLE	Asian	669	ND	ND	ND	2,538	ND	ND	ND	0.044	0.046
Sigurdsson[d], 2005	SLE	Caucasian	480	ND	ND	ND	256	ND	ND	ND	0.180	0.190
Sigurdsson[e], 2005	SLE	Caucasian	109	ND	ND	ND	121	ND	ND	ND	0.160	0.170
Tang, 2015	SLE	Asian	642	352	228	62	672	408	229	35	0.290	0.220

rs280519			**Cases (n) by total and genotype**				**Controls (n) by total and genotype**				**A allele frequency (%)**	
**Author, year**	**Disease**	**Ethnicity**	**Total**	**G/G**	**G/A**	**A/A**	**Total**	**G/G**	**G/A**	**A/A**	**Cases**	**Controls**
Can[a], 2015	CD	Asian	60	20	25	15	89	22	52	15	0.458	0.461
Can[b], 2015	UC	Asian	151	43	63	45	89	22	52	15	0.507	0.461
Graham, 2011	SLE	Caucasian	870	ND	ND	ND	5,551	ND	ND	ND	0.490	0.470
Harley, 2008	SLE	Caucasian	720	ND	ND	ND	2,337	ND	ND	ND	0.530	0.490
Kyogoku, 2009	SLE	Asian	69	19	33	17	94	21	49	24	0.793	0.512
Langefeld, 2017	SLE	Caucasian	3,174	ND	ND	ND	5,464	ND	ND	ND	0.519	0.479
Li, 2011	SLE	Asian	669	ND	ND	ND	2,538	ND	ND	ND	0.357	0.349
Lian, 2013	CD	Asian	80	26	41	13	100	29	48	23	0.325	0.375
Sato[a], 2009	CD	Asian	83	11	35	37	200	39	98	63	0.657	0.560
Sato[b], 2009	UC	Asian	114	ND	ND	ND	200	39	98	63	0.607	0.560
Sigurdsson[d], 2005	SLE	Caucasian	480	ND	ND	ND	256	ND	ND	ND	0.510	0.510

Sigurdsson[e], 2005	SLE	Caucasian	109	ND	ND	ND	121	ND	ND	ND	0.460	0.520
Strange, 2010	Pso	Caucasian	6,748	ND	ND	ND	11,516	ND	ND	ND	0.510	0.470
Tang, 2015	SLE	Asian	642	183	270	189	612	150	256	206	0.510	0.550
rs280523			**Cases (n) by total and genotype**				**Control (n) by total and genotype**				**A allele frequency (%)**	
**Author, year**	**Disease**	**Ethnicity**	**Total**	**G/G**	**G/A**	**A/A**	**Total**	**G/G**	**G/A**	**A/A**	**Cases**	**Controls**
Can[a], 2015	CD	Asian	60	53	7	0	89	76	13	0	0.058	0.073
Can[b], 2015	UC	Asian	151	128	22	1	89	76	13	0	0.079	0.073
Sato[a], 2009	CD	Asian	83	71	12	0	200	170	30	0	0.072	0.075
Sato[b], 2009	UC	Asian	114	ND	ND	ND	200	170	30	0	0.076	0.075
Sigurdsson[d], 2005	SLE	Caucasian	480	ND	ND	ND	256	ND	ND	ND	0.090	0.070
Sigurdsson[e], 2005	SLE	Caucasian	109	ND	ND	ND	121	ND	ND	ND	0.110	0.110
rs2304256			**Cases (n) by total and genotype**				**Controls (n) by total and genotype**				**A allele frequency (%)**	
**Author, year**	**Disease**	**Ethnicity**	**Total**	**C/C**	**C/A**	**A/A**	**Total**	**C/C**	**C/A**	**A/A**	**Cases**	**Controls**
Almlöf, 2017	SLE	Caucasian	1,160	665	442	53	2,711	1,334	1,144	232	0.236	0.297
Alonso-Pérez, 2014	SLE	Caucasian	1,457	ND	ND	ND	1,728	ND	ND	ND	0.234	0.275
Can[a], 2015	CD	Asian	60	11	15	34	89	7	35	47	0.308	0.275
Can[b], 2015	UC	Asian	151	28	38	85	89	7	35	47	0.311	0.275

Contreras-Cubas, 2019	SLE	Mixed	368	ND	ND	ND	516	ND	ND	ND	0.169	0.203
Graciolo[h], 2019	T1DM	Mixed	151	86	57	8	169	98	63	8	0.241	0.233
Graciolo[i], 2019	T1DM	Mixed	156	103	45	8	150	83	60	7	0.195	0.246
Hellquist, 2009	SLE	Caucasian	265	ND	ND	ND	344	ND	ND	ND	0.210	0.310
Järvinen[h], 2010	SLE	Caucasian	169	ND	ND	ND	344	ND	ND	ND	0.240	0.310
Järvinen[i], 2010	SLE	Caucasian	39	ND	ND	ND	344	ND	ND	ND	0.330	0.310
Kyogoku, 2009	SLE	Asian	411	160	180	71	467	175	231	61	0.392	0.378
Li, 2011	SLE	Asian	669	ND	ND	ND	2,538	ND	ND	ND	0.424	0.413
Lian, 2013	CD	Asian	80	38	42	0	100	49	51	0	0.262	0.255
López-Isac[d], 2016	MS	American/ Caucasian	7,103	4,140	2,530	433	12,220	6,499	4,790	931	0.239	0.272
López-Isac[e], 2016	MS	American/ Caucasian	4,985	2,827	1,826	332	7,478	3,848	3,022	608	0.250	0.283
Nagafuchi, 2015	T1DM	Asian	244	103	104	37	254	115	116	23	0.364	0.318
Sato[a], 2009	CD	Asian	83	52	25	6	200	86	90	24	0.233	0.345
Sato[b], 2009	UC	Asian	114	ND	ND	ND	200	86	90	24	0.304	0.345
Sigurdsson[d], 2005	SLE	Caucasian	480	ND	ND	ND	256	ND	ND	ND	0.240	0.320

Sigurdsson[e], 2005	SLE	Caucasian	109	ND	ND	ND	121	ND	ND	ND	0.210	0.270
Sigurdsson, 2007	RA	Caucasian	1,530	ND	ND	ND	861	ND	ND	ND	0.290	0.300
Suarez-Gestal, 2009	SLE	Caucasian	1,546	930	559	57	1,725	903	684	138	0.233	0.278
Suarez-Gestal, 2009	RA	Caucasian	1,635	ND	ND	ND	1,906	ND	ND	ND	0.258	0.269
Tang, 2015	SLE	Asian	642	27	248	367	642	17	160	465	0.210	0.140
Zaplakhova, 2018	MS	Asian	224	142	69	13	312	168	120	24	0.212	0.269

rs12720270			**Cases (n) by total and genotype**				**Controls (n) by total and genotype**				**A allele frequency (%)**	
**Author, year**	**Disease**	**Ethnicity**	**Total**	**G/G**	**G/A**	**A/A**	**Total**	**G/G**	**G/A**	**A/A**	**Cases**	**Controls**
Contreras-Cubas, 2019	SLE	Mixed	368	ND	ND	ND	516	ND	ND	ND	0.156	0.176
Hellquist, 2009	SLE	Caucasian	258	ND	ND	ND	339	ND	ND	ND	0.150	0.220
Järvinen[h], 2010	SLE	Caucasian	159	ND	ND	ND	339	ND	ND	ND	0.190	0.220
Järvinen[i], 2010	SLE	Caucasian	38	ND	ND	ND	339	ND	ND	ND	0.260	0.220
Kyogoku, 2009	SLE	Asian	69	25	31	13	94	34	50	10	0.413	0.372
Li, 2011	SLE	Asian	669	ND	ND	ND	2,538	ND	ND	ND	0.453	0.443
Sigurdsson[d], 2005	SLE	Caucasian	480	ND	ND	ND	256	ND	ND	ND	0.180	0.170
Sigurdsson[e], 2005	SLE	Caucasian	109	ND	ND	ND	121	ND	ND	ND	0.140	0.180
Tang, 2015	SLE	Asian	642	2	95	545	642	2	101	539	0.370	0.360

rs12720356			Cases (n) by total and genotype				Controls (n) by total and genotype				C allele frequency (%)	
**Author, year**	**Disease**	**Ethnicity**	**Total**	**AA**	**A/C**	**C/C**	**Total**	**A/A**	**A/C**	**C/C**	**Cases**	**Controls**
Almlöf, 2017	SLE	Caucasian	1,158	996	154	8	2,711	2,241	444	26	0.073	0.091
Contreras-Cubas, 2019	SLE	Mixed	368	ND	ND	ND	516	ND	ND	ND	0.007	0.023
Diogo[a], 2015	RA	Caucasian	4,726	ND	ND	ND	13,683	ND	ND	ND	0.076	0.084
Diogo[b], 2015	SLE	Caucasian	3,053	ND	ND	ND	13,683	ND	ND	ND	0.075	0.085
Diogo[c], 2015	IBD	Caucasian	1,346	ND	ND	ND	13,683	ND	ND	ND	0.104	0.085
Ellinghaus[a], 2016	CD	Caucasian	19,085	ND	ND	ND	34,213	ND	ND	ND	0.094	0.084
Ellinghaus[b], 2016	Pso	Caucasian	6,530	ND	ND	ND	34,213	ND	ND	ND	0.067	0.084
Ellinghaus[c], 2016	UC	Caucasian	14,413	ND	ND	ND	34,213	ND	ND	ND	0.095	0.084
Harley, 2008	SLE	Caucasian	720	ND	ND	ND	2,711	ND	ND	ND	0.070	0.080
Hellquist, 2009	SLE	Caucasian	277	ND	ND	ND	356	ND	ND	ND	0.070	0.090
Järvinen[h], 2010	SLE	Caucasian	177	ND	ND	ND	356	ND	ND	ND	0.020	0.090
Järvinen[i], 2010	SLE	Caucasian	42	ND	ND	ND	356	ND	ND	ND	0.050	0.090
López-Isac[d], 2016	MS	American/ Caucasian	7,103	6,197	879	27	12,220	10,399	1,759	62	0.066	0.077
López-Isac[e], 2016	MS	American/	4,985	4,347	602	36	7,478	6,356	1,072	50	0.068	0.078

		Caucasian										
Prieto-Pérez, 2015	Pso	Caucasian	191	176	15	0	197	164	30	3	0.037	0.091
Shaiq, 2013	Pso	Asian	351	ND	ND	ND	593	ND	ND	ND	0.009	0.010
Strange, 2010	Pso	Caucasian	3,174	ND	ND	ND	5,464	ND	ND	ND	0.070	0.010
Sigurdsson[d], 2005	SLE	Caucasian	480	ND	ND	ND	256	ND	ND	ND	0.070	0.130
Sigurdsson[e], 2005	SLE	Caucasian	109	ND	ND	ND	121	ND	ND	ND	0.080	0.070
Sigurdsson, 2007	RA	Caucasian	1,530	ND	ND	ND	861	ND	ND	ND	0.090	0.090
rs34536443			**Cases (n) by total and genotype**				**Controls (n) by total and genotype**				**C allele frequency (%)**	
**Author, year**	**Disease**	**Ethnicity**	**Total**	**G/G**	**G/C**	**C/C**	**Total**	**G/G**	**G/C**	**C/C**	**Cases**	**Controls**
Ban, 2009	MS	Caucasian	2,753	ND	ND	ND	1,104	ND	ND	ND	0.035	0.048
Contreras-Cubas, 2019	SLE	Mixed	368	ND	ND	ND	516	ND	ND	ND	0.012	0.021
Couturier, 2011	MS	Caucasian	726	ND	ND	ND	522	ND	ND	ND	0.051	0.096
Diogo[a], 2015	RA	Caucasian	4,726	ND	ND	ND	13,687	ND	ND	ND	0.031	0.043
Diogo[b], 2015	SLE	Caucasian	3,053	ND	ND	ND	13,687	ND	ND	ND	0.024	0.043
Diogo[c], 2015	IBD	Caucasian	1,346	ND	ND	ND	13,687	ND	ND	ND	0.034	0.043
Langefeld[d], 2017	SLE	Caucasian	6,748	ND	ND	ND	11,516	ND	ND	ND	0.022	0.042
Langefeld[e], 2017	SLE	Hispanic	1,872	ND	ND	ND	2,016	ND	ND	ND	0.005	0.019

López-Isac[d], 2016	MS	American/ Caucasian	7,103	6,901	202	0	12,220	11,735	480	5	0.014	0.020
López-Isac[e], 2016	MS	American/ Caucasian	4,985	4,727	257	1	7,478	7,055	409	14	0.026	0.029
Mero[d], 2010	MS	Caucasian	1,112	ND	ND	ND	1,147	ND	ND	ND	0.036	0.042
Mero[e], 2010	MS	Caucasian	810	ND	ND	ND	1,079	ND	ND	ND	0.021	0.031
Mero[f], 2010	MS	Caucasian	1,396	ND	ND	ND	2,064	ND	ND	ND	0.042	0.051
Mero[g], 2010	MS	Caucasian	2,111	ND	ND	ND	1,877	ND	ND	ND	0.032	0.042
Mohamadhosseini, 2019	RA	Asian	700	691	8	1	700	693	7	0	0.017	0.026
Myrthianou[a], 2017	RA	Caucasian	392	385	5	2	507	488	19	0	0.011	0.019
Myrthianou[b], 2017	Pso	Caucasian	126	126	0	0	507	488	19	0	0.000	0.019
Qiu, 2013	MS	Australian	349	ND	ND	ND	498	ND	ND	ND	0.069	0.104
Westra, 2018	T1DM	Caucasian	9,334	ND	ND	ND	11,111	ND	ND	ND	0.030	0.046
rs35018800			**Cases (n) by total and genotype**				**Controls (n) by total and genotype**				**A allele frequency (%)**	
**Author, year**	**Disease**	**Ethnicity**	**Total**	**G/G**	**G/A**	**A/A**	**Total**	**G/G**	**G/A**	**A/A**	**Cases**	**Controls**
Diogo[a], 2010	RA	Caucasian	4,726	ND	ND	ND	13,687	ND	ND	ND	0.0056	0.0076
Diogo[b], 2010	SLE	Caucasian	3,053	ND	ND	ND	13,687	ND	ND	ND	0.0028	0.0076

Diogo[c], 2010	IBD	Caucasian	1,346	ND	ND	ND	13,687	ND	ND	ND	0.0052	0.0076
Ellinghaus[a], 2016	CD	Caucasian	19,085	ND	ND	ND	34,213	ND	ND	ND	0.0052	0.0087
Ellinghaus[b], 2016	Pso	Caucasian	6,530	ND	ND	ND	34,213	ND	ND	ND	0.0045	0.0087
Ellinghaus[c], 2016	UC	Caucasian	14,413	ND	ND	ND	34,213	ND	ND	ND	0.0057	0.0087
López-Isac[d], 2016	MS	American/ Caucasian	7,103	7,046	57	0	12,220	12,053	167	0	0.0040	0.0070
López-Isac[e], 2016	MS	American/ Caucasian	4,985	4,942	43	0	7,478	7,349	127	2	0.0040	0.0080

CD: Crohn's disease; UC: ulcerative colitis; IBD: inflammatory bowel disease; SLE: systematic lupus erythematosus; RA: rheumatoid arthritis; MS: multiple sclerosis; Pso: psoriasis; T1DM: type 1 diabetes mellitus; ND: no data. [a, b, c]: different diseases; [d, e, f, g]: different populations; [h, i]: different samples.
