# Supplementary material for: Association of TYK2 polymorphisms with autoimmune diseases: A comprehensive and updated systematic review with meta-analysis
Source: Genet Mol Biol. 2021 May 3;44(2):e20200425. doi: 10.1590/1678-4685-GMB-2020-0425 (PMC8097517; doi:10.1590/1678-4685-GMB-2020-0425)
Supplement: Table S2 - [file 1415-4757-GMB-44-2-e20200425-s5.pdf]

Genetics and Molecular Biology
https://doi.org/10.1590/1678-4685-GMB-2020-0425

Supplementary Material to "Association of *TYK2* polymorphisms with autoimmune diseases: A comprehensive and updated systematic review with meta-analysis"

Table S2 - Newcastle-Ottawa and Clark-Baudouin quality assessment scale for the studies included in the meta-analysis.

Author	Year	Newcastle-Ottawa Score	Clark-Baudouin Score
Almlöf *et al.*	2017	9	10
Alonso-Pérez *et al.*	2014	8	9
Ban *et al.*	2009	8	9
Can *et al.*	2015	8	9
Contreras-Cubas *et al.*	2019	9	10
Couturier *et al.*	2011	8	9
Diogo *et al.*	2015	9	10
Ellinghaus *et al.*	2016	9	10
Graciolo *et al.*	2019	8	8
Graham *et al.*	2011	7	8
Harley *et al.*	2008	9	9
Hellquist *et al.*	2009	8	9
Järvinen *et al.*	2010	7	8
Kyogoku *et al.*	2009	8	8
Langefeld *et al.*	2017	9	10
Li *et al.*	2011	7	8
Lian *et al.*	2013	6	7
López-Isac *et al.*	2016	8	9

Author	Year	Newcastle-Ottawa Score	Clark-Baudouin Score
Mero *et al.*	2010	8	8
Mohamadhosseini *et al.*	2019	9	9
Myrthianou *et al.*	2017	8	9
Nagafuchi *et al.*	2015	8	8
Prieto-Pérez *et al.*	2015	9	10
Qiu *et al.*	2013	9	10
Sato *et al.*	2009	8	9
Shaiq *et al.*	2013	7	8
Sigurdsson *et al.*	2005	8	9
Sigurdsson *et al.*	2007	8	9
Strange *et al.*	2010	9	9
Suarez-Gestal *et al.*	2009	8	9
Suarez-Gestal *et al.*	2009	9	10
Tang *et al.*	2015	9	9
Westra *et al.*	2018	9	10
Zaplakhova *et al.*	2018	8	9

Quality of articles were calculated using the Newcastle-Ottawa Scale (NOS), as described in the Methods Section. Selection: minimum 1 – maximum 4 stars; Comparability: 0 – 1 star; and Exposure: 1 – 3 stars. In addition, the Clark-Baudouin Score (CBS) was also used to assess the quality of the studies. The CBS consists in a scoring systems that allows from 0 to 10 points regarding pre-defined criteria.
